# Neuroblastoma patient-derived xenograft cells cultured in stem-cell promoting medium retain tumorigenic and metastatic capacities but differentiate in serum

**DOI:** 10.1038/s41598-017-09662-8

**Published:** 2017-08-31

**Authors:** Camilla U. Persson, Kristoffer von Stedingk, Daniel Bexell, My Merselius, Noémie Braekeveldt, David Gisselsson, Marie Arsenian-Henriksson, Sven Påhlman, Caroline Wigerup

**Affiliations:** 1 Translational Cancer Research, Lund University Cancer Center at Medicon Village, Lund University, Lund, Sweden; 2 Department of Pediatrics, Clinical Sciences, Lund University, Lund, Sweden; 3 Department of Clinical Genetics, Lund University, Department of Pathology, University and Regional Laboratories, Lund, Sweden; 4 Department of Microbiology, Tumor and Cell Biology (MTC), Karolinska Institutet, SE-171 77 Stockholm, Sweden

## Abstract

Cultured cancer cells serve as important models for preclinical testing of anti-cancer compounds. However, the optimal conditions for retaining original tumor features during *in vitro* culturing of cancer cells have not been investigated in detail. Here we show that serum-free conditions are critical for maintaining an immature phenotype of neuroblastoma cells isolated from orthotopic patient-derived xenografts (PDXs). PDX cells could be grown either as spheres or adherent on laminin in serum-free conditions with retained patient-specific genomic aberrations as well as tumorigenic and metastatic capabilities. However, addition of serum led to morphological changes, neuronal differentiation and reduced cell proliferation. The epidermal growth factor (EGF) and basic fibroblast growth factor (bFGF) were central for PDX cell proliferation and *MYCN* expression, and also hindered the serum-induced differentiation. Although serum induced a robust expression of neurotrophin receptors, stimulation with their cognate ligands did not induce further sympathetic differentiation, which likely reflects a block in PDX cell differentiation capacity coupled to their tumor genotype. Finally, PDX cells cultured as spheres or adherent on laminin responded similarly to various cytotoxic drugs, suggesting that both conditions are suitable *in vitro* screening models for neuroblastoma-targeting compounds.

## Introduction

Neuroblastoma is a pediatric solid tumor of the sympathetic nervous system with an unmet need of novel treatment approaches for children with high-risk, metastasizing disease^1^. Neuroblastoma is a prototypical tumor type for studying tumor cell differentiation. The overall tumor differentiation stage, as scored by the expression levels of neuronal sympathetic marker genes, strongly correlates to clinical stage and patient outcome, where indolent tumors are generally more differentiated than aggressive tumors^2^. Histopathological assessment of neuroblastoma cell differentiation status is commonly performed as part of the clinical diagnostic procedure^3^ and the differentiating agent isotretinoin is part of standard-of-care therapy for children with high-risk neuroblastoma.

Human cancer cell lines are widely used as preclinical models to test novel drugs for cancer therapy. Despite their historical importance for understanding basic tumor biological questions, it is still uncertain how well cancer cell lines represent the primary tumor^4^. Traditionally, cancer cell lines have been established in serum-containing medium, which seems to select for fast growing cell types that do not fully resemble the *in vivo* situation. Serum-grown cells also differ phenotypically and genetically compared to their original tumor^5, 6^, and *in vivo* models based on xenografted cell lines rarely recapitulate the clinical course seen in patients. Thus, the usefulness of these models to evaluate potential new anti-cancer agents can be questioned, especially if these agents aim to target invasive and metastatic growth. There is a general need for establishing improved *in vitro* and *in vivo* tumor models. Neuroblastoma cell lines established in serum-containing medium have been available for more than 40 years^7^ and they have been essential for molecular characterization of defined aberrant pathways in neuroblastoma. They have also served as models of *in vivo* growth and treatment responses when cultured as xenografts in immune-deficient mice. However, the fact that xenografted neuroblastoma cell lines do not show robust metastatic growth, despite being established from aggressive, metastatic tumors, indicates that they do not fully mimic the tumors they derive from.

Patient-derived xenografts (PDXs), i.e. tumor cells or tissue pieces immediately engrafted in mice without any prior *in vitro* culture step, generally results in tumors that more closely reflect the primary tumors they were derived from as compared to xenografts based on classical cell lines^8, 9^. We recently established and characterized orthotopic neuroblastoma PDXs from high-risk patients and demonstrated that neuroblastoma PDXs maintain and recapitulate patient tumor characteristics^10, 11^. Importantly, the orthotopic PDXs metastasize to clinically relevant sites, including bone marrow^10^. Tumor cells derived from PDXs can further be cultured as spheroids in stem-cell promoting medium with retained tumor-initiating and metastasizing capacity.

Here we report a comprehensive characterization of two *MYCN* amplified neuroblastoma PDX-derived cell lines, named LU-NB-2 and LU-NB-3. The PDX cells were routinely cultured as spheres under conditions initially optimized for growing neural stem cells. The same conditions were recently used for establishing neuroblastoma tumor initiating cells^12^ and here we tested whether serum-free conditions were more optimal for culturing LU-NB-2 and LU-NB-3 cells as compared to serum conditions. We observed that serum induced adherent growth of PDX cells and also sympathetic neuronal differentiation with an accompanied downregulation of *MYCN* expression and activity. Furthermore, serum-culture led to a significant downregulation of TERT complex genes. Spheroid cultures, however, present multiple drawbacks when e.g. screening for drugs; it is labor intensive and cellular heterogeneity can arise due to non-vascularized 3D growth and oxygen/nutrient deficiency in sphere centers. To facilitate future drug screens we therefore investigated conditions promoting monolayer culture, without affecting tumor- initiating and metastasizing capacities. The PDX cells could be grown as monolayer on recombinant human laminin without inducing significant alteration of the phenotype or *in vivo* behavior. The laminin-attached and sphere-cultured PDX cells, respectively, responded similarly to cytotoxic drugs, suggesting that both models are suitable *in vitro* systems for future drug screening.

## Results

### Neuroblastoma PDX cells retain metastatic capacity and patient-specific genomic aberrations following prolonged *in vitro* culturing

**Figure 1:**
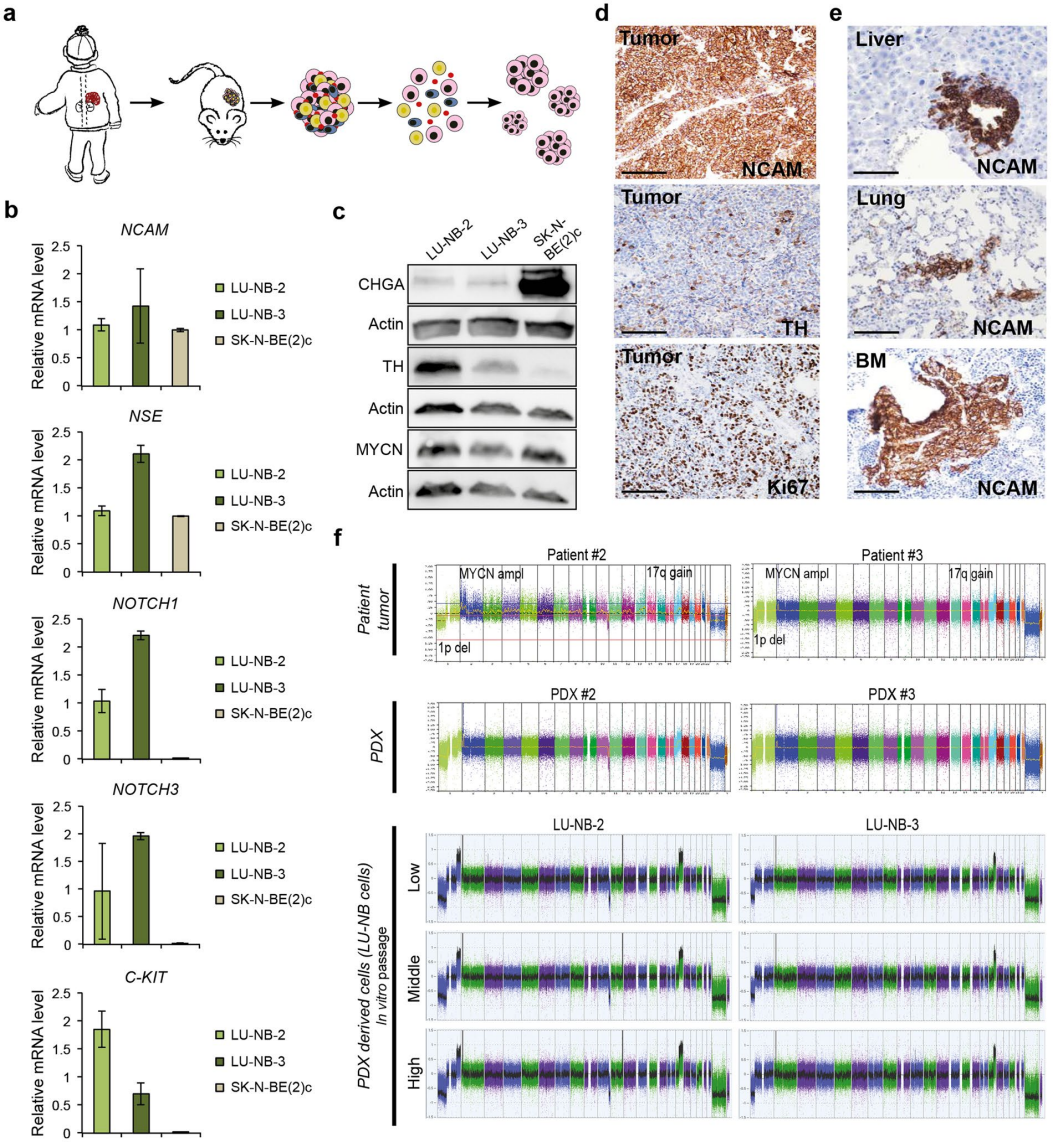
*In vitro* cultured neuroblastoma PDX cells maintain tumorigenic and metastatic capacity *in vivo* and retain patient-specific genomic aberrations. (**a**) Isolation and culture procedure of neuroblastoma PDX cells. (**b**) mRNA expression of neuronal markers (*NCAM* and *NSE*) and stem-cell associated markers (*NOTCH1, NOTCH3* and *C-KIT*) in LU-NB-2, LU-NB-3 and the SK-N-BE(2)c neuroblastoma cell line. (**c**) Western blotting of neuronal markers in LU-NB-2, LU-NB-3 and SK-N-BE(2)c. (**d**) IHC staining of orthotopic PDX cell xenografts (LU-NB-2 or LU-NB-3 cells). Scale bars, 100 μm. (**e**) NCAM staining of metastatic PDX cells in liver, lung and bone marrow (BM) of tumor bearing mice. Scale bars, 100 μm. (**f**) SNP array profiles of patient tumors; patient #2 and patient #3 (upper panels), the corresponding xenografts; PDX #2 and PDX #3 (middle panels), and corresponding PDX cells LU-NB-2 and LU-NB-3 (lower panels). The SNP profiles of patient tumors and PDXs are taken from Braekeveldt *et al*.^10^. Error bars represent ± SEM from 3 independent analyses. *P ≤ 0.05, **P ≤ 0.01, ***P ≤ 0.001; Student’s t test.

Previously, we reported that cells isolated from neuroblastoma PDXs can grow as neurospheres in stem-cell promoting medium (SC medium) (ref. 10 and Fig. 1a). Here we have further characterized cells isolated from PDX #2 and PDX #3, established from high-risk *MYCN* amplified neuroblastomas^10^. The corresponding cell lines LU-NB-2 and LU-NB-3 are thus *MYCN* amplified and express typical neuroblastoma markers, as shown previously^10^ and here by *NCAM* (also known as *CD56*) and *NSE* expression as well as chromogranin A (CHGA) and tyrosine hydroxylase (TH) protein expression, thereby confirming their neuroblastoma origin (Fig. 1a–c). Orthotopic injection of different numbers of LU-NB-2 cells revealed that 10^6^ and 10^4^ cells gave rise to tumors in all mice, whereas 10^2^ cells did not result in a visible tumor 1 year after injection (Supplementary Table S1) (due to ethical reasons, mice could not be kept longer). Orthotopic injection of 10^6^ LU-NB-3 cells gave rise to tumors in 3 out of 4 mice (Supplementary Table S1). PDX cell-derived tumors exhibited strong NCAM staining and heterogeneous expression of TH and Ki67 positive tumor cells (Fig. 1d). Metastasis to lungs, liver and bone barrow were detected in tumor-bearing animals as shown by NCAM staining (Fig. 1e and Supplementary Table S1).

The capacity to seed metastasis is hypothesized to be a trait of cancer stem cells and while neuronal marker expression levels were in parity with those of the classical serum-cultured SK-N-BE(2) c neuroblastoma cells, PDX cells had higher expression of putative neuroblastoma stem-cell associated markers such as *NOTCH1*, *NOTCH3* and *C*-*KIT* (also known as *CD117* or *SCFR*) (Fig. 1b)^13^.

To confirm that cultured PDX cells retained patient specific genomic aberrations we performed single nucleotide polymorphism (SNP) array. This revealed that cultured PDX cells at low (passage 3), middle (passage 15) and high (passage 30) passages retained the genomic aberration profiles (e.g. 1p deletion, *MYCN* amplification and 17q gain) observed in the corresponding patient tumor and xenograft (Fig. 1f). No other gross genomic changes were observed at later passages, suggesting that the PDX cells are genomically stable in long-term culture. Importantly, tumor-initiating and metastasizing capacity was maintained also at high *in vitro* passage (passage 54) (Supplementary Fig. S1). Thus, the neuroblastoma PDX cells retain tumor-initiating and metastatic capacity also after longer *in vitro* passaging.

### Serum promotes differentiation and reduces proliferation

**Figure 2:**
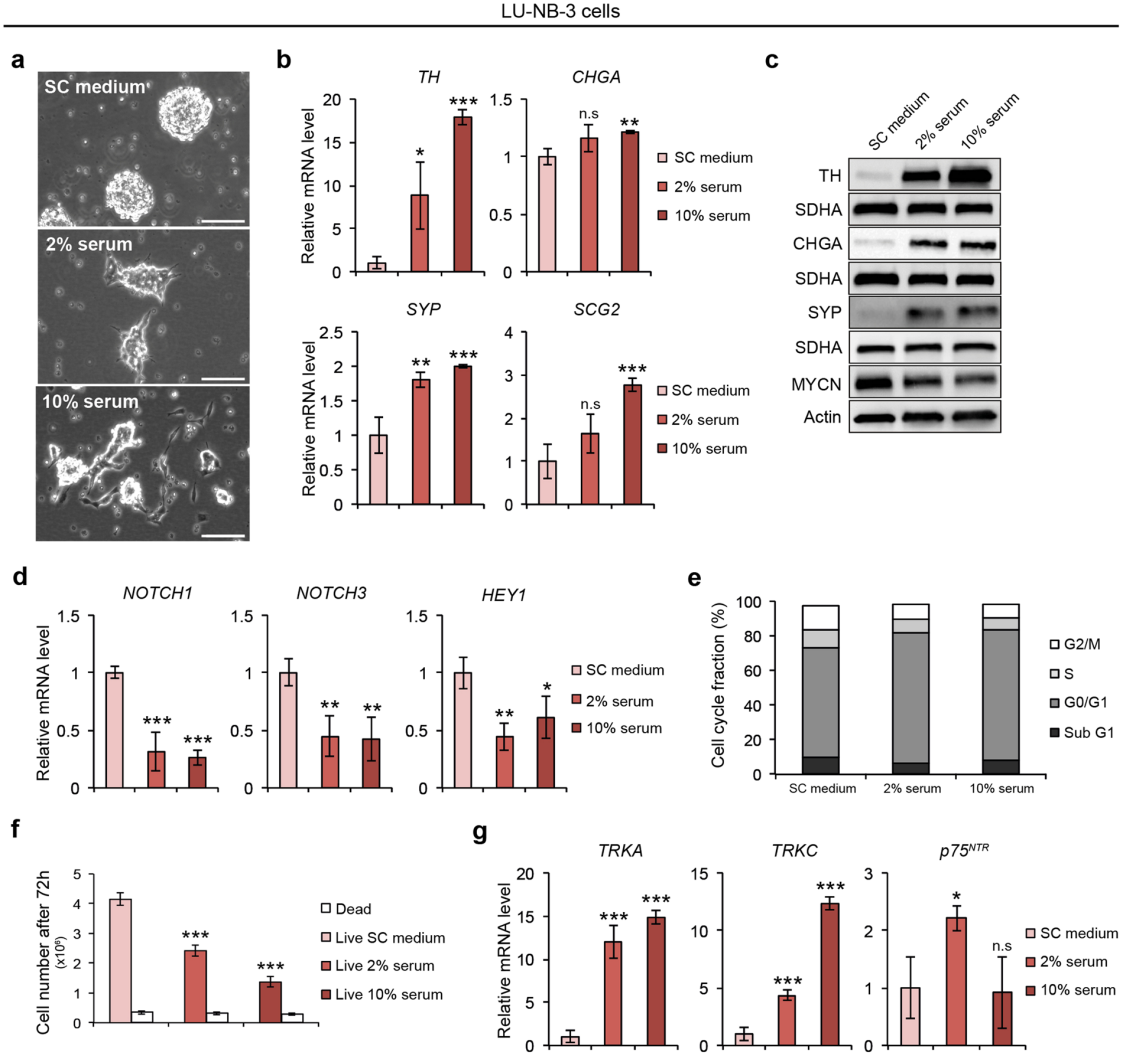
Serum-induced differentiation of PDX cells. LU-NB-3 cells were cultured in stem cell (SC) medium, 2% serum or 10% serum for 7 days unless other specified (**a**–**g**). (**a**) LU-NB-3 cells grown in SC medium (top panel), 2% serum (middle panel) or 10% serum (lower panel). Scale bars, 100 μm. (**b**,**c**) Expression of neuronal markers and MYCN at mRNA (**b**) and protein (**c**) level. (**d**) mRNA expression of markers associated with immature SNS phenotype. (**e**) Cell cycle analysis (72 h). Representative data from 1 experiment is shown (n = 3). (**f**) Live cells (red bars) and dead cells (white bars) determined by trypan blue staining (72 h). (**g**) Expression of neurotrophin receptor genes at the mRNA level. Error bars represent ± SEM from 3 independent analyses. *P ≤ 0.05, **P ≤ 0.01, ***P ≤ 0.001; Student’s t test.

The optimal culture condition for LU-NB-2 and LU-NB-3 cells was explored by comparing short-term serum-grown PDX cells and serum-free cultured cells. Addition of serum induced cell attachment of both LU-NB-2 and LU-NB-3 cells (Fig. 2a and Supplementary Fig. S2a). Other overt serum concentration dependent effects were morphological differentiation and reduced cell numbers (Fig. 2a,f and Supplementary Fig. S2a). The attached cells showed neurite outgrowth and a robust increase in TH expression, both at mRNA and protein level, suggesting serum-driven differentiation (Fig. 2a–c and Supplementary Fig. S2a–c). In addition, mRNA and protein expression of other sympathetic neuronal markers including CHGA, SYP and SCG2^14^, also increased (Fig. 2b,c and Supplementary Fig. S2b,c). The majority of differentiation markers was induced in a serum-concentration dependent manner in both LU-NB-2 and LU-NB-3 cells, however LU-NB-2 cells were less responsive to serum (Fig. 2a–c and Supplementary Fig. S2a–c). This could potentially reflect the fact that PDX #2 was established from relapsed disease while PDX #3 was established prior to treatment. The expression of genes associated with an immature sympathetic nervous system phenotype, e.g. *NOTCH1*, *NOTCH3* and *HEY1* was downregulated in response to serum (Fig. 2d). Furthermore, serum led to an increased number of cells in G0/G1 phase at the expense of S/G2/M phase cells (Fig. 2e). There was no increase in the sub G0/G1 fraction as determined by flow cytometry, indicating that the reduced cell number in serum was not due to cell death, a result corroborated by trypan blue staining (Fig. 2f). Continued growth in 10% serum for up to 6 weeks revealed that the cells survived, were still differentiated but with stagnating growth rates (Supplementary Fig. S2d,e). Taken together, LU-NB-2 and LU-NB-3 cells cultured under serum-free conditions maintain a less differentiated, proliferative cell phenotype while serum reduces proliferation and induces neuronal differentiation.

The neurotrophins NGF and NT-3 and their cognate receptors, TrkA and TrkC, are required for terminal differentiation of proliferating, non-transformed sympathetic neuroblasts^15, 16^. In neuroblastoma, high TrkA and TrkC expression is associated with low stage and less aggressive disease^17, 18^. The *TRKA* (also known as *NTRK1*) and *TRKC* (also known as *NTRK3*) expression increased robustly in serum-cultured LU-NB-3 cells (Fig. 2g), further supporting our conclusion that serum induces sympathetic differentiation. The obligatory co-receptor p75^NTR^ subunit of high affinity neurotrophin receptors is expressed in these cells, although at low levels (Fig. 2g). Interestingly, treating serum-cultured LU-NB-3 cells with NT-3 or NGF alone or in combination did not further increase the expression of *TH*, *DBH* or *SCG2* (Supplementary Fig. S2f) nor did it enhance the serum-induced morphological differentiation. Cultured neuroblastoma cells expressing *TRKA* or *TRKC* are not resistant to neurotrophin stimulation, per se, as exemplified by NGF or NT-3 treated *TRKA* or *TRKC* overexpressing SH-SY5Y cells, although neurothrophin treatment in those cells did not induce terminal differentiation^19, 20^. In line with reported data obtained from established neuroblastoma cell lines^21^, we propose that also the PDX cells lack the capacity to terminally differentiate.

### MYCN, telomerase complex genes and neuroblastoma PDX cell differentiation

**Figure 3:**
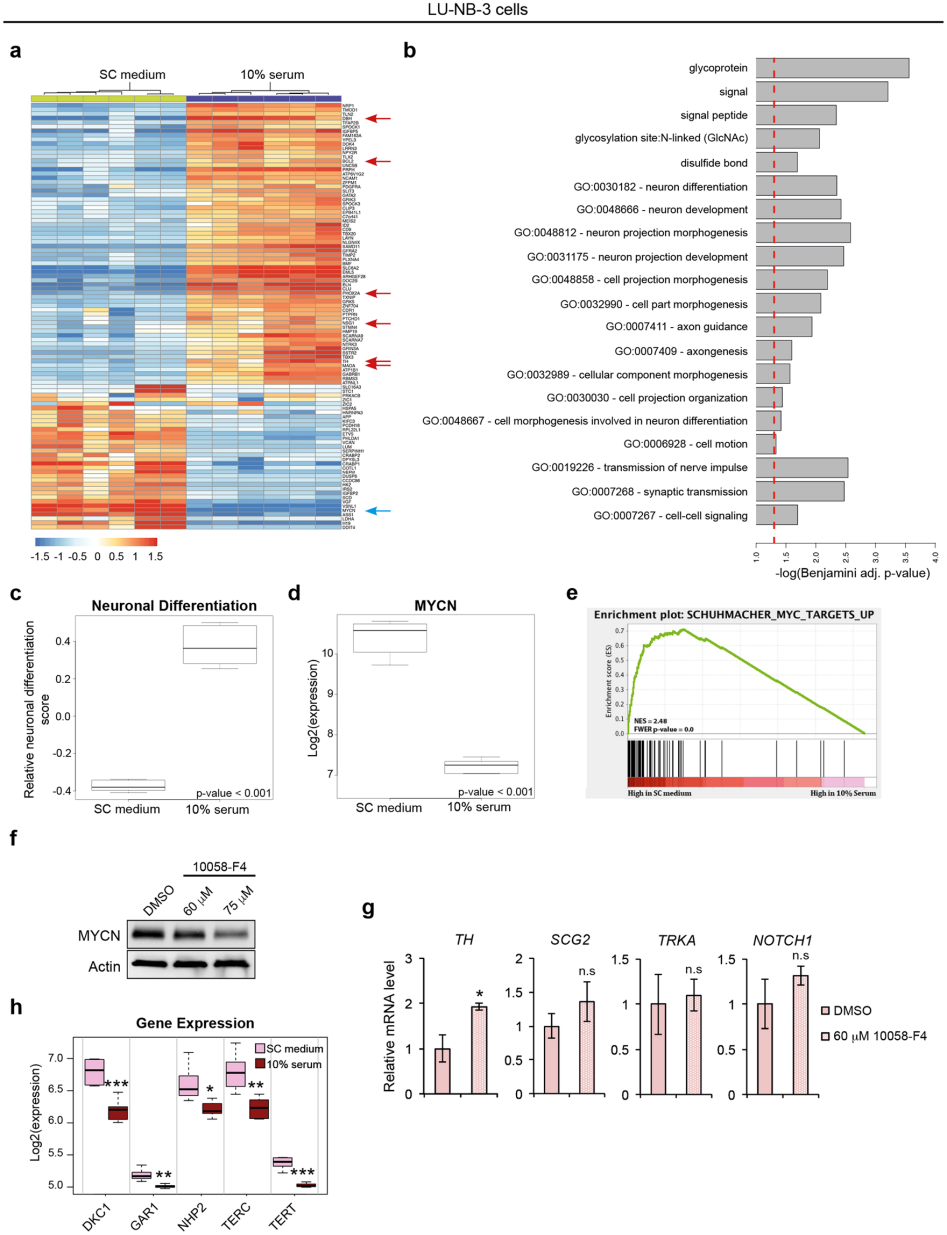
Serum promotes neuronal development, downregulation of MYCN activity and telomerase complex components in PDX cells. LU-NB-3 cells were cultured in stem cell (SC) medium or 10% serum for 7 days unless other specified. (**a**) Heatmap showing the top 100 varying genes across all samples. (**b**) Gene ontology analysis showing significantly enriched terms from the top 3 ontology clusters enriched in 10% serum cultured LU-NB-3 cells. Dashed line indicates Benjamini p-value < 0.05 (full list of ontologies provided in Supplementary File S1). (**c**,**d**) Boxplots of a neuronal differentiation score based on Fredlund *et al*.^2^ (**c**) and *MYCN* expression levels (**d**). (**e**) GSEA of MYCN target genes based on a ranked gene list of all genes according to differential expression between the two culture conditions. (**f**) MYCN western blot after 10058-F4 treatment (72 h). (**g**) mRNA expression of differentiation markers and *NOTCH1* after 10058-F4 treatment (72 h). (**h**) Boxplots showing expression of telomerase complex proteins (*DKC1, GAR1* and *NHP2*) as well as the RNA subunit *TERC* and the enzymatic subunit *TERT*, in different culture conditions. Error bars represent ± SEM from 3 independent experiments. *P ≤ 0.05, **P ≤ 0.01, ***P ≤ 0.001; Student’s t test.

RNA sequencing of LU-NB-3 cells cultured in either serum-free or serum-containing medium revealed a general induction of a sympathetic neuronal phenotype at serum conditions as exemplified by increased expression of *DBH*, *TH*, *PHOX2A*, *BCL2*, *MAOA* and *NSG1* (Fig. 3a). Furthermore, gene ontology analysis revealed a significant enrichment of neuronal differentiation in the samples of serum-cultured cells (Fig. 3b,c and Supplementary File S1). Interestingly, *MYCN* was the top downregulated gene in serum-cultured LU-NB-3 cells, showing over 9-fold decrease in mRNA expression level (Fig. 3d). This result was further supported by decreased protein levels (Fig. 2c and Supplementary Fig. S2c). Gene set enrichment analysis (GSEA) revealed a significant enrichment of genes regulated by *MYCN* in serum-free cultured cells (Fig. 3e and Supplementary File S2).

*MYCN* inhibition has previously been proposed to promote neuroblastoma cell differentiation, and we therefore treated LU-NB-3 cells with the MYC-MAX inhibitor 10058-F4. Treatment with 10058-F4 led to decreased MYCN protein, verifying MYCN inhibition (Fig. 3f). At mRNA level, treatment with 60 μM 10058-F4 for 72 h led to a modest upregulation of *TH* but had little or no effect on *SCG2*, *TRKA* and *NOTCH1* expression, indicating that MYCN inhibition promotes differentiation of neuroblastoma PDX cells to a limited extent (Fig. 3g).

Tumor cells are characterized by high proliferation and are dependent on active telomerase to circumvent shortening of chromosomal ends. Because MYC can induce *TERT* expression^22^ we asked whether downregulation of *MYCN* and reduced proliferation in serum was coupled to decreased expression of *TERT*. Interestingly, *TERT* and other components of the telomerase complex, including *TERC*, *DKC1*, *GAR1* and *NHP2*, were all downregulated in serum-grown LU-NB-3 cells (Fig. 3h). The snoRNPs DKC1, GAR1 and NHP2 have been shown to be important for telomerase activity in neuroblastoma^23^. A signature score based on these markers correlates with segmental aberrations and is an independent predictor of poor prognosis in neuroblastoma^23, 24^.

### The SC medium growth factors EGF and bFGF decrease serum-induced differentiation

**Figure 4:**
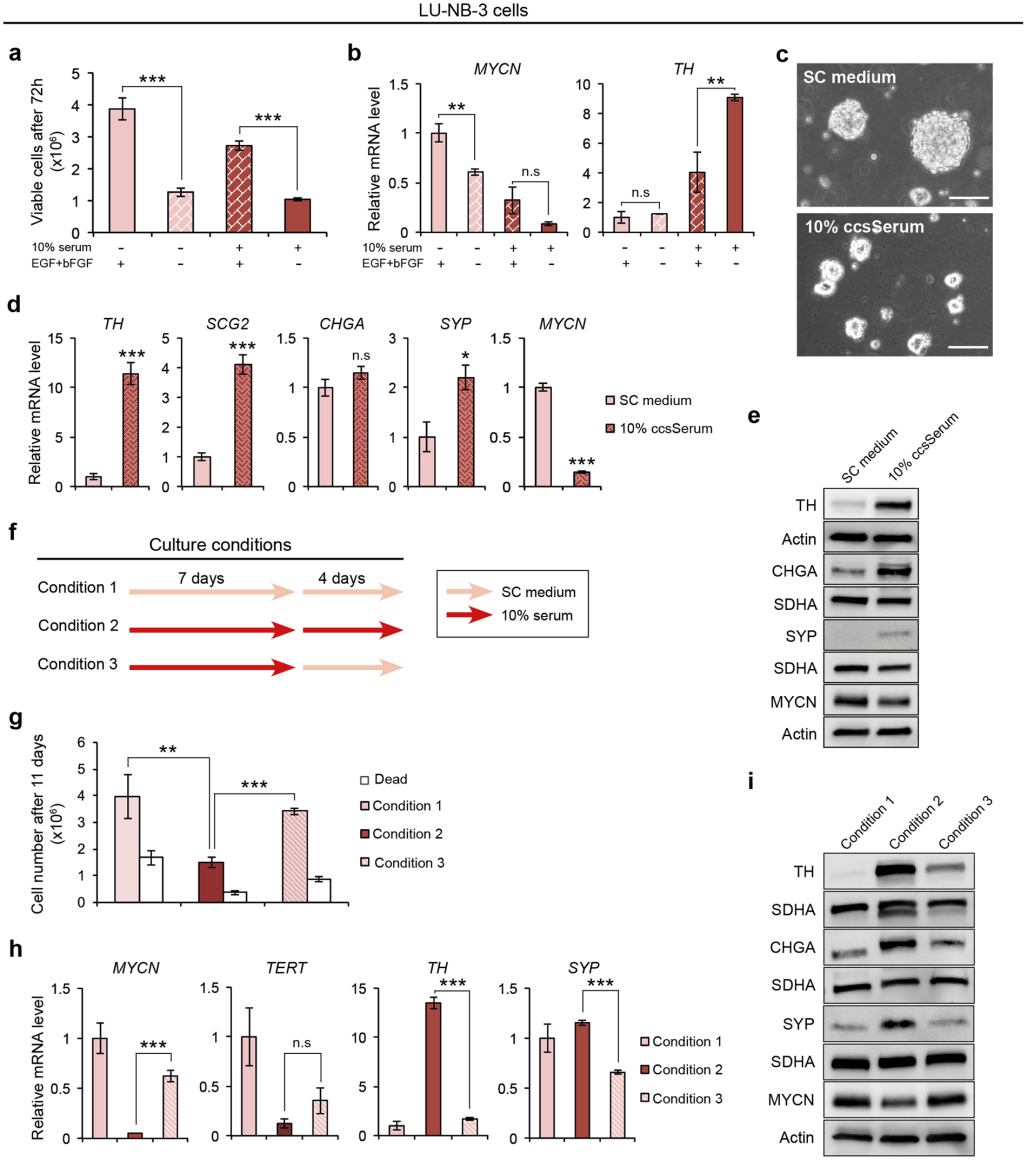
Serum-induced differentiation of PDX cells is suppressed by stem cell growth factors and is reversible. (**a**,**b**) LU-NB-3 cells grown in presence of serum or growth factors as indicated. (**a**) Live cells determined by trypan blue staining (72 h). (**b**) mRNA expression of *MYCN* and *TH* levels. (**c**) LU-NB-3 cells grown in stem cell (SC) medium or 10% charcoal-stripped serum (ccsSerum). Scale bars, 100 μm. (**d**,**e**) mRNA (**d**) and protein (**e**) expression of neuronal markers and MYCN. (**f**–**i**) The serum-induced differentiation is reversible. (**f**) Culture conditions for LU-NB-3 cells. (**g**) Live (red bars) and dead cells (white bars) determined by trypan blue staining after culture conditions specified in (**f**). (**h**) mRNA expression of *MYCN* *,* *TERT* and neuronal markers after culture conditions specified in (**f**). (**i**) Western blotting of neuronal markers after culture conditions specified in (**f**). Error bars represent ± SEM from 3 independent experiments. *P ≤ 0.05, **P ≤ 0.01, ***P ≤ 0.001; Student’s t test.

For serum culture conditions we excluded the growth factors EGF and bFGF. These are two important components of stem-cell promoting medium previously shown to support expansion of neural stem cells by symmetrical division^25^. Withdrawal of EGF and bFGF from SC medium led to lower cell numbers and lowered *MYCN* levels in LU-NB-3 cells (Fig. 4a,b). However, this did not induce differentiation as determined by *TH* expression levels (Fig. 4b). Furthermore, addition of EGF and bFGF to serum-containing medium led to a less pronounced differentiating effect of added serum (Fig. 4b). Thus, our data suggest that EGF and bFGF are important for sustained proliferation of neuroblastoma PDX cells and that these factors diminish serum-induced differentiation.

Glucocorticoid receptor signaling has been reported to promote differentiation of neuroblastoma cells^26^. Since serum contains glucocorticoids we tested if 10% lipid-free, charcoal-stripped serum would induce differentiation. This condition did not promote cellular attachment or morphological differentiation, yet the expression of differentiation markers was still induced and *MYCN* levels decreased (Fig. 4c–e). We hypothesized that charcoal-stripped serum might still contain protein-bound corticosteroids able to induce the expression of differentiation markers and therefore measured the amount of steroids in regular and charcoal-stripped serum. Charcoal-stripped serum was principally free of steroids (Supplementary Table S2), suggesting that glucocorticoid signaling is not responsible for upregulation of differentiation markers at serum culture conditions.

### Serum-induced differentiation of PDX cells is reversible

As we did not observe further differentiation by neurothrophin treatment we asked whether the serum-induced differentiation was reversible. LU-NB-3 cells were cultured in 10% serum medium for 7 days and then transferred back to SC medium for 4 days (Fig. 4f). This led to an increased proliferation and *MYCN* levels, and reduced expression of *TH* and *SYP* (Fig. 4g–i). Thus, the cellular differentiation induced by serum is reversible, which is in line with the observation that the PDX cells could not be terminally differentiated.

We next asked whether neuroblastoma cells that have been cultured in serum for decades would convert to a less differentiated phenotype in serum-free medium. We explored this by growing SK-N-BE(2)c cells for 7 and 14 days in either standard serum-containing or SC medium. SK-N-BE(2)c cells cultured in SC medium presented with a less flattened morphological appearance and grew in clusters, but not as spheres (Supplementary Fig. S3a). Furthermore, differentiation marker expression data did not uniformly suggest that these cells acquired a less differentiated phenotype (Supplementary Fig. S3b).

### Laminin promotes adherent growth and maintains the tumorigenic and metastasizing phenotype

**Figure 5:**
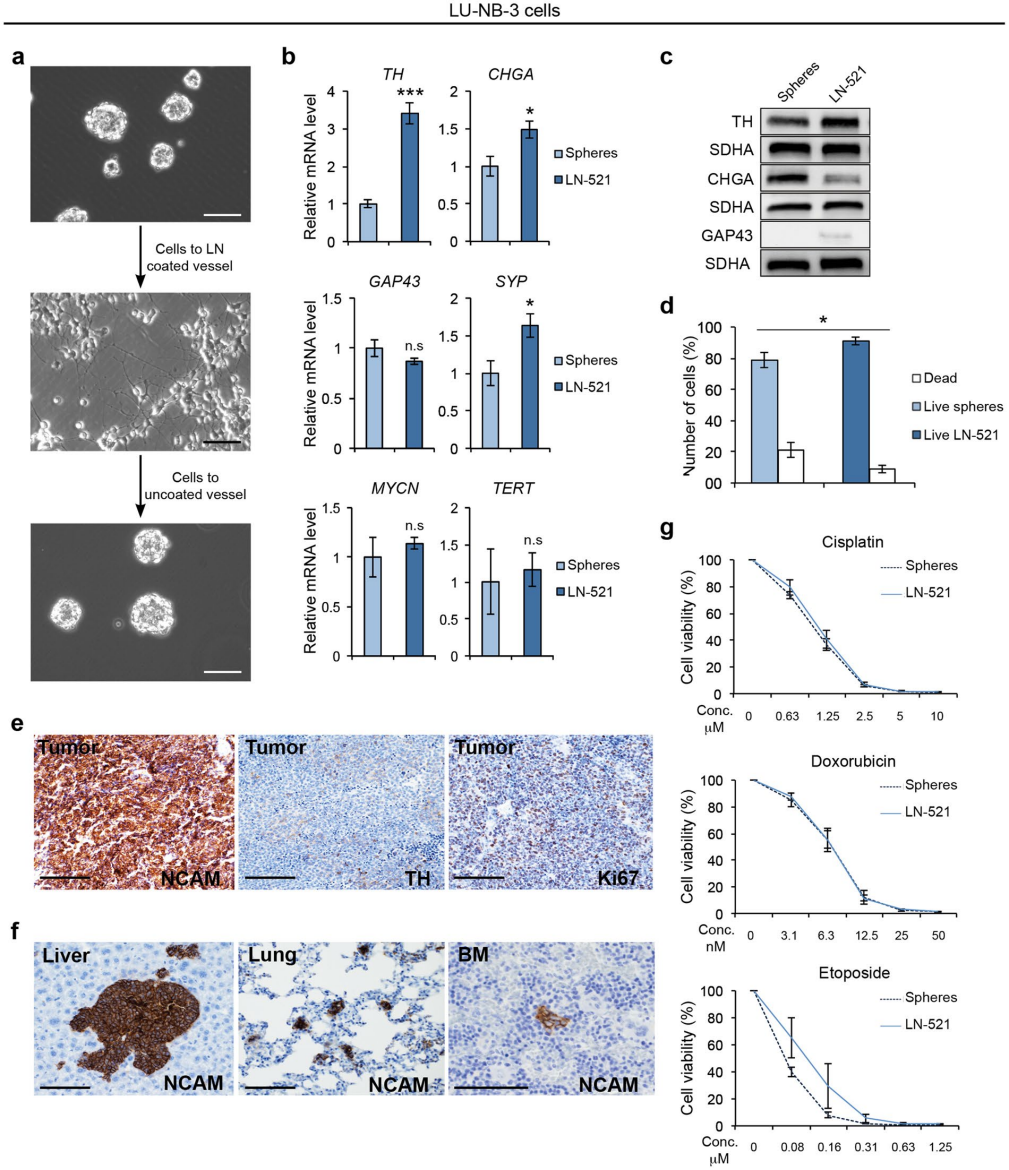
Laminin promotes viability of neuroblastoma PDX cells and maintains tumorigenic and metastasizing capacity without inducing differentiation. LU-NB-3 cells were grown as spheres in stem cell (SC) medium or adherently on laminin (LN-521) for 72 h (**a**–**g**). (**a**) Cells grown as spheres in SC medium (top panel), adherently on LN-521 (middle panel) and as spheres after laminin-growth (lower panel). Scale bars, 100μm. (**b**,**c**) Expression of neuronal markers, *MYCN* and *TERT* at mRNA level (**b**) and protein level (**c**) in LU-NB-3 cells. (**d**) Live (blue bars) and dead cells (white bars) as determined by trypan blue staining of sphere or laminin cultures (72 h). (**e**,**f**) IHC staining of tumors (**e**) and metastases to the liver, lung and bone marrow (BM) (**f**) formed by injected long-term LN-521-cultured LU-NB-3 cells. Scale bars, 100 μm. (**g**) Cell viability of sphere or LN-521-cultured LU-NB-3 cells after 72 h treatment with cisplatin, doxorubicin and etoposide at various concentrations. Drugs were added 24 h after seeding. Error bars represent ± SEM from 3 independent experiments. *P ≤ 0.05, **P ≤ 0.01, ***P ≤ 0.001; Student’s t test.

As serum-induced adherence resulted in strong phenotypic changes we sought alternative protocols to grow neuroblastoma PDX cells as a monolayer. Adherently grown glioma, as well as mouse and human neural stem cells, have successfully been established on laminin-1 (laminin α1-β1-γ1) with retained stemness features^25, 27, 28^. The neuroblastoma PDX cells, however, adhered poorly to this substrate. A screening of laminin isoforms synthetized by the neuroblastoma PDX cells revealed that laminin α5 chain was highly expressed in both LU-NB-2 and LU-NB-3 (Supplementary Fig. S4a). We therefore tested recombinant human laminin 511 (LN-511) and 521 (LN-521) as substrates for the PDX cells. Though both laminin isoforms promoted adherent growth of LU-NB-2 and LU-NB-3, we used LN-521 for further experiments. Following adherent growth on LN-521, PDX cells again formed spheres when transferred back to uncoated dishes (Fig. 5a). As laminin α5 was significantly upregulated in serum-grown LU-NB-3 cells (Supplementary Fig. S4b) and PDX cells adhering to LN-511 or LN-521 showed signs of morphological differentiation, we asked whether surface adherence alone could induce a differentiated phenotype. PDX cells grown on LN-521 showed a modest increase in *TH*, *CHGA* and *SYP* expression (Fig. 5b and Supplementary Fig. S4c) as compared to serum-grown PDX cells (e.g. compare 3.4-fold increase in *TH* expression in Fig. 5b and 17.9-fold increase in Fig. 2b). At the protein level, TH and GAP43 levels slightly increased in LN-521-cultured LU-NB-2 and LU-NB-3 cells, while CHGA was downregulated, despite its increased mRNA expression (Fig. 5c and Supplementary Fig. S4d). In addition, the expression of genes associated with a less differentiated phenotype was not affected by laminin growth (Supplementary Fig. S4e). Monolayer culture provides uniform access to growth factors and LN-521-cultured LU-NB-3 cells were more viable than sphere-grown cells (Fig. 5d). *MYCN* expression did not differ between LN-521-cultured and sphere-cultured LU-NB-3 cells (Fig. 5b), although it was slightly lower in LN-521-cultured LU-NB-2 cells (Supplementary Fig. S4c). Importantly, *TERT* levels were unaffected in LN-521-cultured PDX cells (Fig. 5b and Supplementary Fig. S4c). In conclusion, neuroblastoma PDX cells can grow adherently on laminin, which promotes the viability of the cells with little effect on differentiation. As anticipated, addition of serum to LN-521-cultured cells promoted differentiation (Supplementary Fig. S5a,b).

To determine whether PDX cells cultured on LN-521 maintained tumor-initiating and metastatic capacities, we cultured cells on LN-521 for 3 months before injecting cells orthotopically into the adrenal gland of NSG mice. All animals (n = 6) developed tumors with typical neuroblastoma morphology and examination of lungs, liver and bone marrow revealed metastatic growth in all examined animals (Fig. 5e,f). Finally, the response to various cytotoxic drugs did not differ between sphere- and LN-521-cultured cells (Fig. 5g and Supplementary Fig. S4f), suggesting that laminin adherent cells are promising models for future drug screening.

## Discussion

Conventional tumor-derived cell lines established decades ago, including neuroblastoma cell lines, have traditionally been cultured in the presence of serum. Although derived from highly aggressive and metastatic tumors they show limited infiltrative growth in rodent xenograft models and generally do not metastasize. We previously established and characterized orthotopic neuroblastoma PDX models and showed that PDX tumors infiltrated adjacent tissues and seeded metastases to liver, lungs, and bone marrow. Such patterns of infiltrative growth and metastases are not observed with orthotopically xenografted conventional cell lines^10^. We further showed that neuroblastoma cells isolated from PDXs could be cultured short-term as spheres, and when re-injected they formed tumors and metastases^10, 11^. Here, we show that these cells retain their proliferative capacity after long-term passaging, suggesting that they have infinite *in vitro* growth capacity. The PDX cell lines retain patient-specific genomic aberrations up to at least 30 passages without obtaining additional gross DNA aberrations. Furthermore, their tumorigenic and metastatic capacities are retained also at high *in vitro* passages. We further demonstrate that the sphere-forming PDX cell lines can be grown as a monolayer on LN-521 without compromising their capacities to form tumors and metastases. Importantly, PDX cells adhering to LN-521 did not significantly alter the cellular response to cytotoxic drugs. As the neuroblastoma PDX tumors are established from high-risk tumors and metastasize to clinically relevant sites, including the bone marrow, the neuroblastoma PDX model offers a highly promising *in vivo* model for pre-clinical drug testing. We further conclude that PDX cells grown as spheres or on LN-521 are suitable *in vitro* systems for future screening of drugs targeting aggressive neuroblastoma.

Further analysis of PDX cells grown on LN-521 revealed that they were slightly more viable and expanded readily as compared to sphere-grown cells, suggesting that laminin-integrin signaling supports neuroblastoma PDX cell survival. Intriguingly, the PDX cells readily attached to two laminin isoforms containing the α5 chain, laminin 511 and 521; the same type of laminins used for growing embryonal stem cells^29, 30^. This may suggest that the neuroblastoma PDX cells share features with cells of embryonal origin. Recent work comparing glioma cells grown as neurospheres versus those grown on laminin, showed no significant differences in growth rate, apoptosis, differentiation markers or tumorigenicity^31^. These findings together with our results support that both growth conditions are suitable for stable expansion of neural-derived tumor cells *in vitro*.

**Figure 6:**
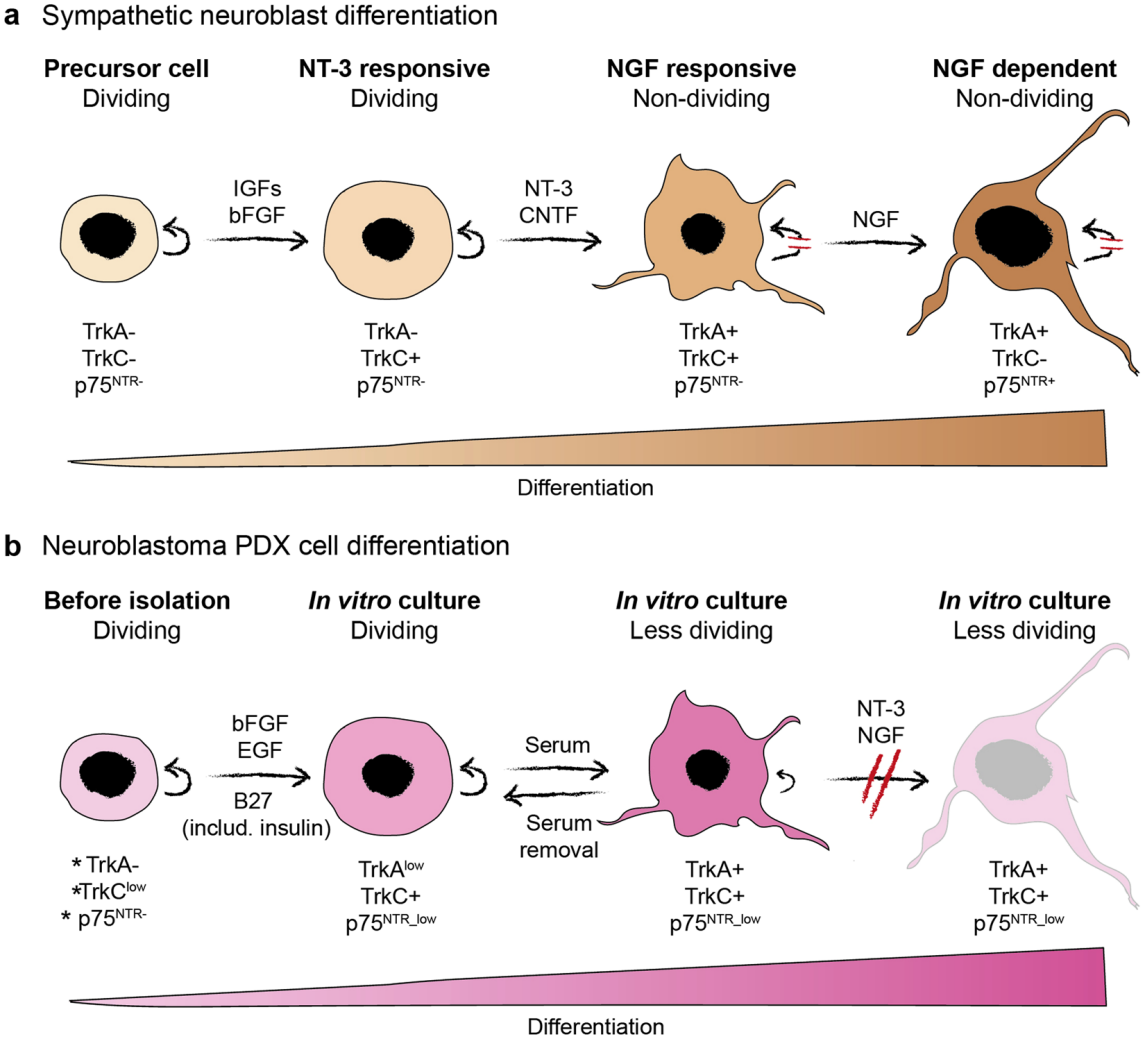
Schematic figure showing growth factor driven differentiation of (**a**) sympathetic neuroblasts (adapted from ref. 16) and (**b**) neuroblastoma PDX cells. (**a**) Sympathetic precursor cells rely on various growth factors such as IGFs, bFGF and CNTF to develop neurotrophin dependency. The neurotrophin receptors TrkA, TrkC and p75^NTR^ are expressed in a sequential manner and cells become dependent on NT-3 and NGF for survival. (**b**) Expression of neurotrophin receptors in PDX tumor tissue (indicated by *) and in *in vitro* cultured PDX cells. Addition of serum to cultured PDX cells resulted in increased expression of *TRKA* and *TRKC*, which was reversed upon serum removal. Treatment with NT-3 and/or NGF in combination with serum did not result in further differentiation. Arched arrows indicate cell self-renewal.

We found that withdrawal of EGF and bFGF and addition of serum also promoted attachment of the PDX cells but led to differentiation, reduced *MYCN* levels and decreased proliferation. Thus, avoiding serum appears to be a key strategy to preserve the original immature features of these neuroblastoma cells which is in line with previously established primary neuroblastoma cells propagated in serum-free media^12^. Differentiation is highly associated with neuroblastoma patient outcome, and it has been proposed that malignant transformation of neuroblasts at different stages during fetal development causes the great phenotypic diversity of this disease^32^. The enigmatic spontaneous differentiation observed in stage 4S neuroblastomas, together with experimentally induced *in vitro* differentiation of neuroblastoma cells^21^ raised hope that aggressive neuroblastomas could be treated by triggering neuronal differentiation. In the clinic, high-risk neuroblastoma patients are treated with the differentiating agent isotretinoin to combat minimal residual disease, however whether the effect of this treatment is due to induced tumor cell differentiation in patients has not been demonstrated. Furthermore, so far, there are no studies demonstrating that neuroblastoma cells in culture established from high-risk patients can terminally differentiate (i.e. acquire an irreversibly differentiated phenotype and ceased proliferation capacity). Here we show that the serum-induced overt morphological differentiation accompanied by increased and robust expression of marker genes is reversible. Furthermore, despite the serum-differentiated PDX cells expressing the neurotrophin receptors TrkC and TrkA, which mediate the key responses leading to terminally differentiated sympathetic neurons^16^, no additive effect was observed when combining serum with the neurothrophins NT-3 and NGF. We conclude that the neuroblastoma PDX cells can be pushed to a more differentiated phenotype; however, they appear to have lost the capacity to terminally differentiate (Fig. 6).

Prolonged culture of LU-NB-3 cells in serum did not result in a regained high growth rate. Whether the PDX cells could be established as classical cell lines in serum-containing medium when cultured for longer periods remains to be shown. Although it would be of academic interest to try to establish PDX cells in serum-medium to compare geno- and phenotypes with PDX cells cultured in SC medium, such cell lines would be of less value for understanding the biology of neuroblastoma. Based on our own observations and on similar findings reported in glioma^5^, we expect that such cells will phenotypically and genotypically deviate from the tumors they were derived from. Theoretically, serum-grown cells experience cellular crisis similar to what happens in tumor cells upon loss of telomerase activity. Reduced telomerase activity in neuroblastoma cells resulted in increased frequency of anaphase-bridges^23^. Thus, upon re-activation of telomerase activity and recovered proliferative capacity, long-term serum-grown cells have likely gained additional genetic aberrations, created by breakage-fusion-bridge cycles.

Considering the significance of *MYCN* in neuroblastoma, our observation that *MYCN* was the top downregulated transcript in serum-grown PDX cells is intriguing. As well as being essential for normal neurogenesis^33^, *MYCN* has been shown to prevent neuronal differentiation^26, 34, 35^, although overexpression of *MYCN* in non-*MYCN*-amplified SK-N-SH cells did not impede differentiation induced by several different protocols^36^. Treatment with the MYC-MAX inhibitor 10058-F4 promoted expression of a few, but not all of the differentiation markers tested, showing that MYCN inhibition alone cannot substantially push the PDX cells towards a more differentiated stage. We speculate that presence of EGF and bFGF hinders differentiation to some extent. It would therefore be interesting to test 10058-F4 without the addition of growth factors. However, withdrawal of growth factors in itself leads to downregulation of *MYCN*, making it difficult to draw conclusions from that type of experiment.

It was recently shown that *MYCN*-regulated miRNAs inhibit the expression of several nuclear hormone receptors, including the glucocorticoid hormone receptor, and simultaneous inhibition of *MYCN* and activation of glucocorticoid signaling resulted in differentiation of neuroblastoma cells^26^. Our data show that charcoal-stripped serum devoid of steroids promoted *MYCN* downregulation and increased the expression of differentiation markers in the PDX cells, however it did not promote attachment or morphological differentiation.

In conclusion, we show that neuroblastoma PDX cells grown under serum-free conditions maintain patient-specific genomic aberrations over time and that tumorigenic and metastatic capabilities, as well as response to cytotoxic drugs, are preserved also at higher *in vitro* passages or when cells are cultured as monolayer on laminin. Our data and other studies showing serum-induced differentiation of tumor cells^5, 37^ strongly suggest that serum should be avoided in order to maintain tumor cell immaturity in culture. The phenotypic effects of serum have mostly been overlooked but they can explain why serum-established cell lines do not metastasize or grow invasively in xenograft models. This observation could account for the generally poor translation of results from pre-clinical screening based on classical cell lines to clinical testing of drugs^4^.

## Materials and Methods

### Neuroblastoma PDX cells, cell lines and cell culture treatments

Tissue from neuroblastoma PDXs was dissociated and digested for 45 min at 37 °C with Liberase (0.15 mg/mL, Roche), passed through a 70 µm cell strainer (BD Biosciences) and cultured in stem cell medium (SC medium) at 37 °C in 5% CO_2_^10^. Spheres were dissociated using Accutase (Sigma-Aldrich, St. Louis, MO). PDX #2 had been established from cerebral metastasis from a stage 4 tumor where the patient had undergone prior treatment while PDX #3 had been established from a primary stage 3 tumor in the adrenal gland. For serum culture, SC medium excluding growth factors and B-27 supplement was supplemented with fetal bovine serum (FBS) or 10% charcoal-stripped FBS (Thermo Scientific). For monolayer culture on human recombinant laminin (Biolamina), plates were coated according to manufacturers instructions. PDX cells were routinely grown to confluence and dissociated using Accutase. The neuroblastoma cell line SK-N-BE(2)c (ATCC) was cultured as previously described^38^. All cells were routinely screened for mycoplasma and authentication was performed by SNP profiling of SK-N-BE(2)c cells, PDX cells and corresponding PDX (Multiplexion, Germany).

Treatment with MYC-MAX inhibitor 10058-F4 (Sigma) was performed for 72 h at 60 μM or 75 μM. Treatment with neurotrophins was done for 4 days with nerve growth factor (NGF, 50 ng/ml, PeproTech), neurotrophin-3 (NT-3, 50 ng/ml, PeproTech), or a combination. Prior to neurotrophin treatment, cells were cultured in SC medium, 2% or 10% serum for 7 days. For cytostatic treatments, cells were treated with cisplatin, doxorubicin or etoposide in BRAND*plates*® 96-well plates (VWR) directly after seeding or 48 h post-seeding. CellTiter-Glo Luminescent Cell Viability Assay (Promega) was used to determine cell viability 72 h post-treatment. Cell culture images were captured with a Carl Zeiss AxioCam IC microscope and the ZEN software.

### Quantitative real-time PCR

Total RNA was extracted using the RNeasy Micro Kit (Qiagen) or the RNeasy Mini Kit (Qiagen). Complementary DNA and qRT-PCR was performed as described previously^38^. Three housekeeping genes (*SDHA*, *UBC*, *YWHAZ*) were used to normalize gene expression. Primer sequences are given upon request.

### Western blotting

Cells were lysed in RIPA buffer supplemented with complete protease inhibitor. Proteins were separated by SDS-PAGE and transferred to HyBond-C-Extra nitrocellulose membranes. The following antibodies were used: CHGA (M0869, Dako), TH (Ab112, Abcam), N-MYC (Sc-791, Santa Cruz BioTechnology), SYP (M0776, Dako), GAP43 (8945, Cell Signaling), Actin (691001, MP Biomedicals), and SDHA (Ab14715, Abcam).

### Cell cycle analysis

Cell cycle distribution was determined by fixating PDX cells in 70% ice-cold ethanol in −20 °C, washed in PBS and incubated on ice for 45 min in Vindelöv solution (3.5 µmol/L Tris-HCl (pH 7.6), 10 mmol/L NaCl, 50 µg/mL propidium iodide, 20 µg/mL RNase, 0.1% v/v NP40). Samples were run on a FACSVerse instrument (BD Biosciences) and data was analyzed using FlowJo software (FlowJo, LLC).

### Single nucleotide polymorphism array

DNA was extracted from PDX cells at low (p3), middle (p15) and high (p30) *in vitro* passages using DNeasy Blood and Tissue Kit (Qiagen). For analysis, genomic DNA was hybridized to an Affymetrix CytoScan HD chip (Affymetrix) containing approximately 2.6 million markers of which almost 750,000 are SNPs. Constitutional copy number variants were removed by filtering against the Database of Genomic Variants (Oct. 2016). Copy number variation analyses were performed using ChAS software. The analyses of xenografts and the corresponding patient tumors were reported previously^10^.

### RNA sequencing and data analysis

PDX cells were cultured in SC medium or 10% serum for 7 days. Total RNA was extracted using the RNeasy Mini Kit (Qiagen) and RIN values were checked using an Agilent 2100 Bioanalyzer (Agilent). RNA-Seq analysis was performed on an Ion Proton System for next-generation sequencing (Thermo Fischer). For each of the samples, 10 ng of total RNA was reverse transcribed using the Ion AmpliSeq Transcriptome Human Gene Expression kit (Revision A.0) following the protocol of the manufacturer (Thermo Fisher) and as previously described^39^.

Sequences were aligned to hg19 AmpliSeq Transcriptome ERCC v1 and were quantified on gene level. Gene level reads were normalized according to total number of reads per sample. Data was analyzed using R statistical language (version 3.1.1). Genes with zero variance were removed from the data. The neuronal differentiation signature score was calculated as described by Fredlund and colleagues^2^. Differential expression was determined using unpaired t-test analysis in R using the ‘stats’ package (version 3.3.2). Gene Ontology (GO) analyses were performed on the top 250 upregulated genes in serum treated samples, using the Database for Annotation, Visualization and Integrated Discovery (DAVID) v6.8 tool^40^. A complete gene list of all genes present in the RNA sequencing data was used as the experimental background for GO analyses. Gene set enrichment analyses (GSEA)^41^ was performed on a ranked list of all genes based on differential expression (t-statistic) between SC and 10% serum treatments using the c2.all.v5.0 curated gene set collection (Broad Institute MSigDB).

### Animal procedures

Four- to six-week-old female or male NSG mice were purchased from Charles River (Charles River Laboratories). Mice were housed under pathogen-free conditions and received autoclaved water and food. Orthotopic injections of PDX cells were performed as previously described^10^. All animal procedures followed the guidelines set by the Malmö-Lund Ethical Committee for the use of laboratory animals and were conducted in accordance with European Union directive on the subject of animal rights. Experimental protocols were approved by the Malmö-Lund Ethical Committee (ethical permits M146-13 and M11-15). The establishment of neuroblastoma PDXs was approved by the regional ethical review board at Lund University (Dnr. 2011/289) and written informed consent was obtained.

### Immunohistochemistry

Xenograft tumors and mice organs were fixed in formalin and bone specimens were decalcified in 10% EDTA (pH 8). Following paraffin embedding, 4 μm tissue sections were stained using AutostainerPlus (Dako). The following antibodies were used: NCAM (Leica Biosystems, NCL-L-CD56-504), Ki67 (MIB-1, Dako), and TH (Ab112, Abcam). Images were acquired using Olympys BX63 microscope and DP80 camera along with the CellSense Dimension imaging software.

### Statistical analysis

All values are reported as mean ± SEM from at least three independent experiments unless otherwise stated. The two-sided Student unpaired *t* test was used for statistical analyses, and three levels of significance were used: *P < 0.05; **P < 0.01; ***P < 0.001.

### Data Availability

All data generated or analyzed during this study are included in this published article (and its Supplementary Information files).

## Electronic supplementary material

Supplementary information

Supplementary file S1

Supplemetary file S2
