# Supplementary material for: Neuroblastoma patient-derived xenograft cells cultured in stem-cell promoting medium retain tumorigenic and metastatic capacities but differentiate in serum
Source: Sci Rep. 2017 Aug 31;7:10274. doi: 10.1038/s41598-017-09662-8 (PMC5579187; doi:10.1038/s41598-017-09662-8)
Supplement: Supplementary file 1 — Supplementary information [file 41598_2017_9662_MOESM1_ESM.pdf]

Supplementary figures and table

Neuroblastoma patient-derived xenograft cells cultured in stem-cell promoting medium retain tumorigenic and metastatic capacities but differentiate in serum

Camilla U. Persson[1], Kristoffer von Stedingk[2], Daniel Bexell[1], My Merselius[1], Noémie Braekeveldt[1], David Gisselsson[3], Marie Arsenian-Henriksson[4], Sven Påhlman[1] and Caroline Wigerup[1]

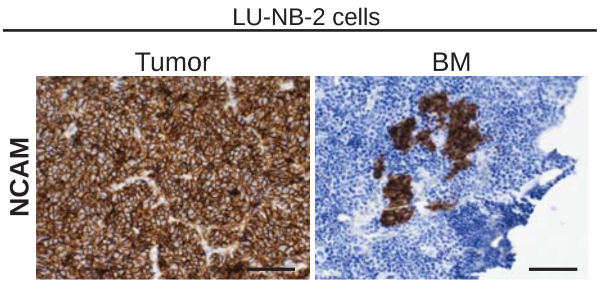

Supplementary Figure S1.
Long-term in vitro cultured PDX cells retain tumor-initiating and metastatic capacity. IHC staining of tumor and bone marrow (BM) tissue from a tumor-bearing mouse after being injected with LU-NB-2 cells cultured for 54 passages. Scale bar, 100 µm.

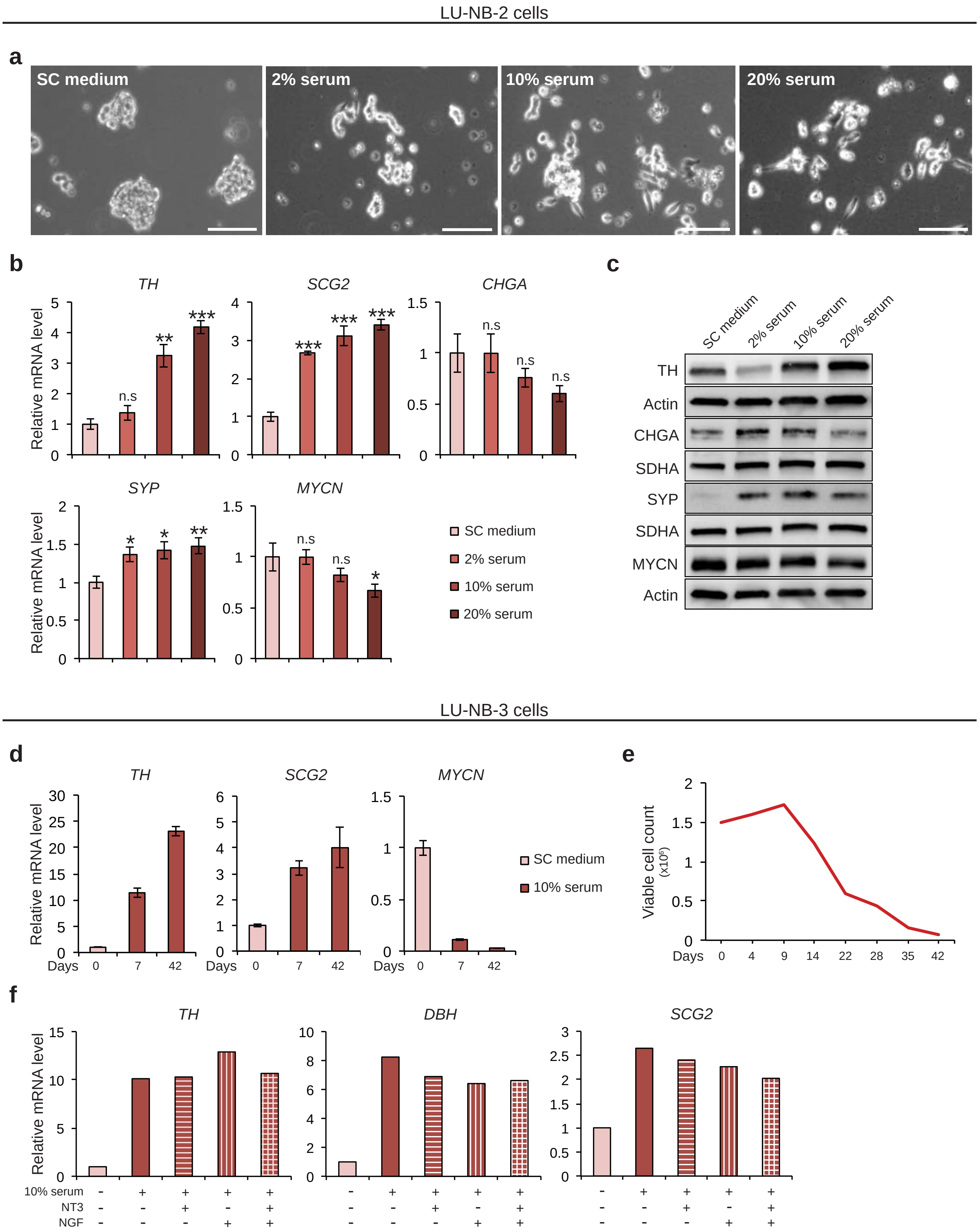

Supplementary Figure S2.

Serum-induced differentiation of PDX cells. (**a**) Morphology of LU-NB-2 cells grown in stem cell (SC) medium, 2% serum, 10% serum or 20% serum for 7 days. (**b-c**) Expression of neuronal markers and MYCN at mRNA level (**b**) and protein level (**c**) after 7 days. (**d**) mRNA expression of neuronal markers and MYCN in LU-NB-3 cells cultured in 10% serum for 6 weeks. (**e**) Live cells determined by trypan blue staining during passage of long-term 10% serum cultured LU-NB-3 cells. (**f**) mRNA expression of neuronal markers in LU-NB-3 cells cultured in 10% serum for 7 days followed by treatment with 50 ng/ml NT3 or 50 ng/ml NGF alone or in combination for 4 days. Mean from two experiments is shown (n=2). Error bars represent ±SEM from 3 independent experiments. *P ≤ 0.05, **P ≤ 0.01, ***P ≤ 0.001; Student's t test.

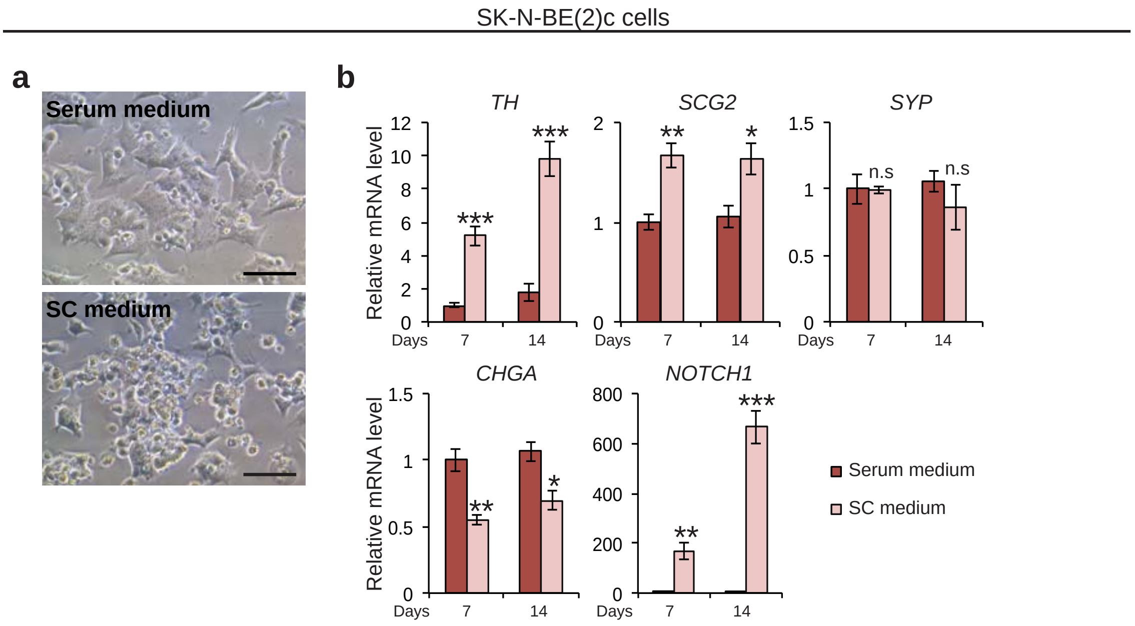

Supplementary Figure S3.
SK-N-BE(2)c cells cultured in stem cell medium. (**a**) SK-N-BE(2)c cells cultured in serum-containing medium (top panel) and serum-free stem cell (SC) promoting medium (lower panel) for 7 days. (**b**) mRNA expression of neuronal markers and NOTCH1 after 7 and 14 days. Error bars represent ±SEM from 3 independent experiments. *P ≤ 0.05, **P ≤ 0.01, ***P ≤ 0.001; Student’s t test.
.

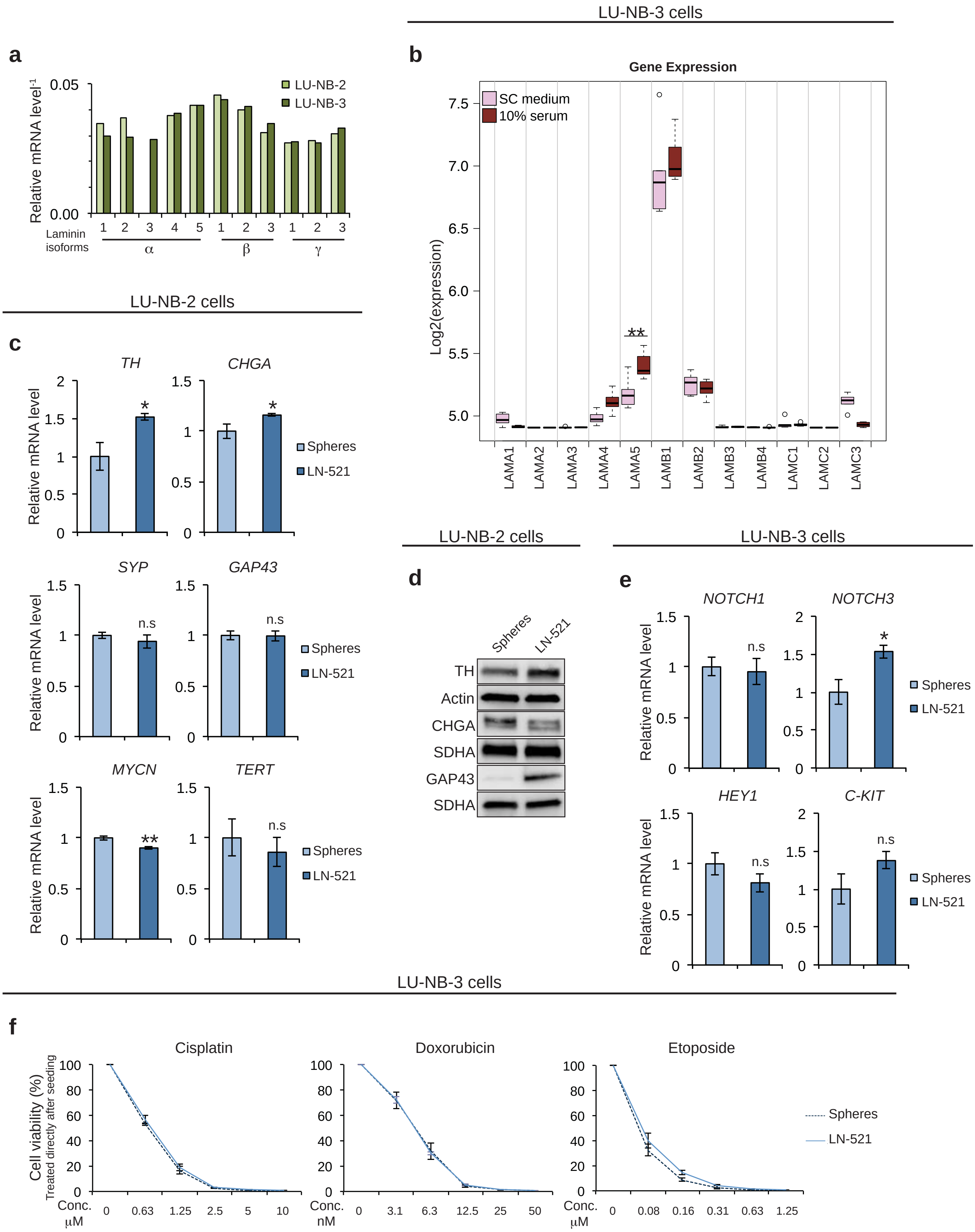

Supplementary Figure S4.

Laminin isoform expression in PDX cells and laminin vs. sphere cultured PDX cells. (**a**) mRNA expression of various laminin chain isoforms in LU-NB-2 and LU-NB-3 cells. (**b**) Serum-induced expression of laminin α5 chain isoform in LU-NB-3 cells (7 days). (**c-d**) Expression of neuronal markers at mRNA level (**c**) and protein level (**d**) in LU-NB-2 cells cultured as spheres or on LN-521 (72h). (**e**) mRNA expression of genes associated with less differentiated phenotype in sphere vs. LN-521-cultured LU-NB-3 cells (72h). (**f**) Cell viability of sphere or laminin cultured LU-NB-2 cells after 72h treatment with cisplatin, doxorubicin and etoposide at various concentrations. Drugs were directly after seeding of cells. Error bars represent ±SEM from 3 independent experiments. *P ≤ 0.05, **P ≤ 0.01, ***P ≤ 0.001; Student's t test.

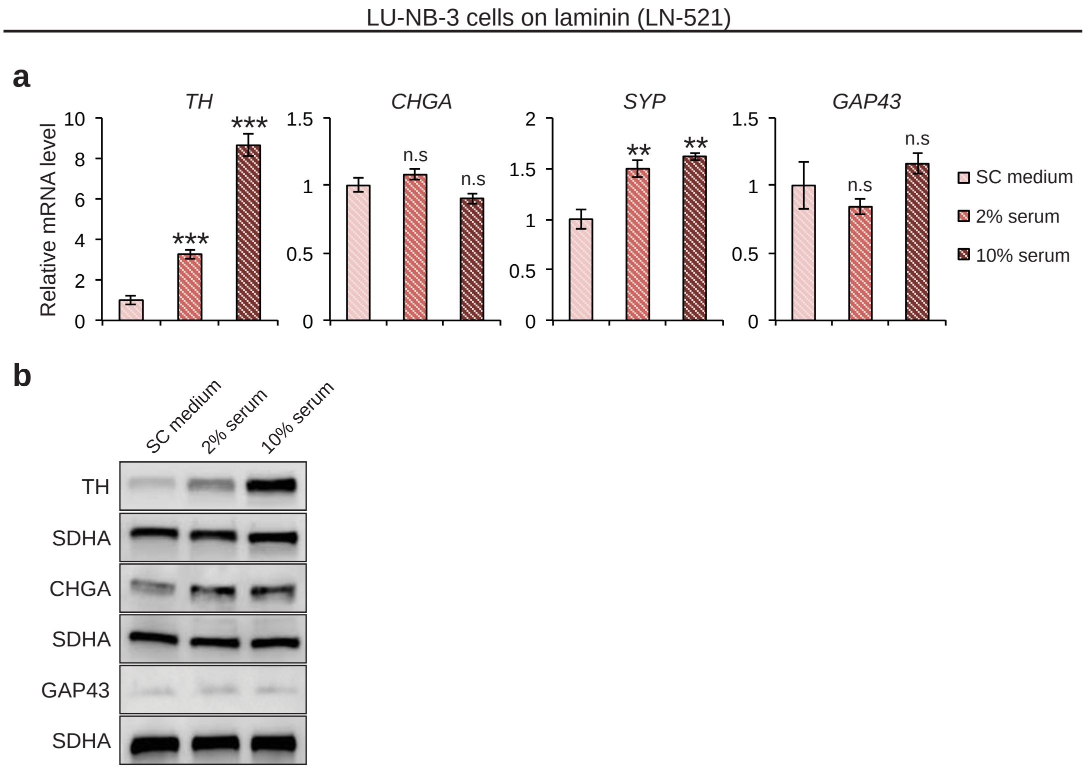

Supplementary Figure S5.

Serum-induced differentiation of laminin-cultured LU-NB-3 cells. Cells were seeded on LN-521 in either stem cell (SC) medium or medium containing 2% or 10% serum for 72h. (**a-b**) Expression of neuronal markers at mRNA (**a**) and protein level (**b**). Error bars represent ±SEM from 3 independent experiments unless other specified. *P ≤ 0.05, **P ≤ 0.01, ***P ≤ 0.001; Student's t test.

Table S1. Orthotopic injections of LU-NB-2 and LU-NB-3 cells in NSG mice.

PDX cells	Number of cells	Mice (n)	Tumor	Days	Metastases		
					Lung	Liver	BM
LU-NB-2	1×10^6	3	Yes (3/3)	118	Yes	Yes	Yes
	1×10^4	4	Yes (4/4)	156 - 172	Yes	Yes	N.D
	1×10^2	4	No (0/4)	-	-	-	-
LU-NB-3	2×10^6	4	Yes (3/4)*	85-100	Yes	Yes	N.D

N.D = not determined
* One mouse died prior to sacrifice and preservation of material.

Table S2. Steroid profiling of fetal bovine serum (FBS) and charcoal-stripped FBS.

Steroid (ng/ml)	FBS	Charcoal-stripped FBS
Aldosterone	n.d.	n.d.
Androstenedione	0.014	n.d.
Corticosterone	0.064	n.d.
Cortisol	2.080	n.d.
Cortisone	0.778	0.002
11-Deoxycorticosterone	n.d.	n.d.
11-Deoxycortisol	0.002	n.d.
DHEA	n.d.	0.261
DHEAS	9.910	n.d.
17OH-Progesterone	0.006	n.d.
Progesterone	n.d.	n.d.
Testosterone	0.083	n.d.
Pregnenolone	0.289	n.d.
21-Deoxycortisol	0.007	0.007
18OH- Cortisol	0.179	n.d.

n.d.=not detected
